# The Scourges: Why Abortion Is Even More Morally Serious than Miscarriage

**DOI:** 10.1093/jmp/jhad014

**Published:** 2023-04-15

**Authors:** Calum Miller

**Affiliations:** University of Oxford, Oxford, UK

**Keywords:** abortion, death, miscarriage, respect, wrongness of killing

## Abstract

Several recent papers have suggested that the pro-life view entails a radical, implausible thesis: that miscarriage is the biggest public health crisis in the history of our species and requires radical diversion of funds to combat. In this paper, I clarify the extent of the problem, showing that the number of miscarriages about which we can do anything morally significant is plausibly much lower than previously thought, then describing some of the work already being done on this topic. I then briefly survey a range of reasons why abortion might be thought more serious and more worthy of prevention than miscarriage. Finally, I lay out my central argument: that reflection on the wrongness of killing reveals that the norms for ending life and failing to save life are different, in such a way that could justify the prioritization of anti-abortion advocacy over anti-miscarriage efforts. Such an account can also respond to similar problems posed to the pro-lifer, such as the question of whom to save in a “burning lab” type scenario.

## I. INTRODUCTION

In recent years, pro-lifers have been criticized for focusing disproportionately on the issue of abortion, while at the same time neglecting the apparently much greater number of deaths from spontaneous abortion (Ord, 2008; Berg, 2017; Simkulet, 2017). A similar problem is suggested by the scenario which asks us to consider whether we would save multiple embryos or a single child in a burning laboratory—the intuition commonly being that we should save the single child.^1^ Different implications might be drawn from this, including that pro-lifers do not *really* believe that embryos are human beings with a right to life,^2^ or that pro-lifers are more interested in limiting women’s autonomy, or both. In this paper, I marshal a series of arguments to explain why these conclusions are unwarranted. In particular, I suggest that reflection on the wrongness of killing shows that it is largely unrelated to consequences, and even largely unrelated to the badness of death. This furnishes the pro-lifer with a reason to seek the prevention of abortion more urgently than the prevention of miscarriage, and indeed for pro-lifers to preferentially rescue older children over embryos. But, it also supplies a more general reason to think that the distinction between killing and letting die is relevant, and for thinking that the norms shaping behavior in those situations differ.

## II. HOW MANY INDUCED ABORTIONS AND MISCARRIAGES ARE THERE?

It is worth briefly reviewing the evidence regarding induced abortion and miscarriage. This does not make a great difference to my central argument, but most authors have noted that even if there are clear reasons to prioritize tackling a lesser number of abortions over a greater number of miscarriages, there is some residual (indeed, substantial) reason for pro-lifers to address those miscarriages which do occur, which undoubtedly number many millions. Let me say something about this.

Estimates for the number of miscarriages vary, although it appears likely that most are exaggerated. Berg suggests up to 89% of all pregnancies, while Ord suggests that there are 226 million worldwide every year. These are almost certainly vast overestimates. In a recent comprehensive review, Jarvis (2017) proposes somewhere between 40% and 60% of all pregnancies end in miscarriage, noting that higher figures are implausibly excessive. Around 10%–35% of embryos are lost before implantation, a further 10%–20% before clinical recognition of pregnancy, and 5%–15% between clinical recognition and birth. We note in particular the large range for embryo loss before implantation: the relevant events are extremely difficult to measure, and so we have very low confidence in our estimates.

Globally, there are around 140 million births a year (Our World in Data, 2020). If around half of the pregnancies are ended prematurely (the midpoint of 40%–60%), then there are around 140 million spontaneous abortions a year. This is obviously a very large number—far exceeding the number of induced abortions, even. That said, it is still 80 million or so less than Ord’s estimate.

This does not, of course, take into account induced abortions. Some pregnancies end neither in birth nor spontaneous abortion, and this will affect the estimate of spontaneous abortions (if there are more total pregnancies, then there are likely more spontaneous abortions, although some of these will result in induced abortions before a miscarriage occurs). This does not affect the estimate by a very great deal, relatively speaking. Very likely, the number is somewhere between 100 and 200 million, which is enough for the argument to get going. Ord and Berg have likely overestimated it by quite some way, however, which reduces considerably the ostensible disproportion with which they allege pro-lifers to act.

Blackshaw and Rodger (2019) note that about a third of spontaneous abortions are anembryonic pregnancies, where the embryo proper does not actually form (or forms briefly, and then disappears). If the embryo never forms, then according to the standard pro-life view, there is no organism and hence, no life is lost. This would reduce the salience of spontaneous abortion for pro-lifers. However, the events in early pregnancy are too poorly understood (at least in frequency) to know how many anembryonic pregnancies involve embryos that formed and then were destroyed, which would presumably still count as deaths. Although there are other “pregnancies” where a human being likely does not form,^3^ I shall ignore these and take the estimate of 140 million lives lost as broadly correct—my argument is precisely intended to explain how pro-lifers are justified in paying more attention to a more limited number of lives.

Although Ord and Berg overestimate the number of miscarriages, the number of abortions is also likely to be substantially lower than some of their pro-life respondents (and authorities such as the WHO) have suggested. Blackshaw and Rodger cite a study from the Guttmacher Institute which estimates around 56 million abortions a year. (Sedgh et al., 2016) Although details of the data used in the Guttmacher study are sparse, it seems reasonable to believe they have relied at least in part on previous Guttmacher studies that overestimate the number of illegal abortions by an order of magnitude in many countries. For example, Koch, Bravo et al. (2012) have provided powerful arguments that the Guttmacher Institute overestimates the number of abortions in Colombia by around 10-fold. In Mexico, a Guttmacher study estimated 137–194,000 abortions per annum for Mexico’s Federal District. After abortion was legalized in this region (the only region in the country), however, the subsequent 5 years yielded only 78,544 abortions across the entire period (Koch, Aracena et al., 2012).^4^ Given how prone to radical overestimates abortion statistics are, 56 million may be a large overestimate—although there are certainly many millions every year.^5^ Probably, the total number of spontaneous abortions still exceeds the number of induced abortions by several times. For Colgrove (2021) and Blackshaw and Rodger (2019), this does affect the argument, although not by a great deal and likewise for my own argument.

## III. CAUSES OF MISCARRIAGE

As Colgrove describes at length, “spontaneous abortion” or “miscarriage” (used interchangeably here) is not a cause of death in itself: it is just the phenomenon of natural deaths in utero (prior to viability, after which point the terminology used is “stillbirth”). So, saying that spontaneous abortion is responsible for more deaths than induced abortion is like saying that natural death is responsible for more deaths than homicide or genocide. This is evidently not a very persuasive argument for not paying significant attention to homicide and genocide, nor is it very informative about the causes of death.

Spontaneous abortion is made up of a variety of different causes, as helpfully summarized by Blackshaw and Rodger. They note that around 60% are due to aneuploidies, where an embryo has an anomalous number of chromosomes in each cell (as in Down Syndrome).^6^ We can also add to these euploid genetic causes (Colley et al., 2019), and hence, the number of miscarriages due to genetic causes is greater—though we are unsure by how much—than simply the proportion due to aneuploidies. Other causes include immunological conditions, thrombophilias, endocrinological causes, uterine malformations, and acute maternal infections. Certain lifestyle factors (e.g., smoking), chronic conditions (e.g., diabetes), and non-modifiable risk factors (e.g., increasing age) also appear to contribute.

Blackshaw and Rodger use this analysis to argue that induced abortion is actually one of the largest causes of prenatal death: on their analysis, 44% are due to aneuploidies, 27% due to induced abortion, and 29% due to other causes.^7^ They note, further, that treating aneuploidies is very difficult since we have no treatment and, since they usually occur before detection of pregnancy, prevention is likewise difficult. Ord rightly objects that this does not rule out research. For example, Alzheimer’s is largely unpreventable, and so we invest instead primarily in research. Ord also suggests sperm sorting to prevent the anomalies in the first place. Blackshaw and Rodger respond to the suggestion of research by noting that most such research is likely to be ethically problematic if, for example, it involves research on embryos. But, in Ord’s defence, it is not entirely clear that this is the case: aneuploidy and spontaneous abortion both occur in animals, and animal models may shed considerable light on the causes and possible prevention. Blackshaw and Rodger note also that keeping human beings with fatal chromosomal defects alive for an inevitably short period is not necessarily an overriding moral obligation: this is true, but if those fatal chromosomal defects are themselves remediable in principle, then this response will not work, since we would have at least some obligation (even if not overriding) to extend their lives.

There is a bit more we can say about the relevance of chromosomal anomalies, however. First, the aneuploidies are themselves quite varied: Hassold et al. (1980) report that about a quarter are due to Turner Syndrome (only one sex chromosome, X), nearly half due to trisomies (such as Down, Patau, and Edwards Syndrome), and nearly a quarter triploidy and tetraploidy (one or two extra copies of *each* chromosome), inter alia.

Here is one pro-life response: just as “miscarriage” is an artificially large group, so too is “aneuploidy”: it groups together a wide range of conditions (from Down Syndrome to Turner Syndrome, to tetraploidy), which do not really constitute a single cause of death. The largest cause of death is Turner Syndrome, responsible for, we suppose, 15% of spontaneous abortions, so perhaps 20,000,000 lives lost a year. This may be equal to, or even fewer than, the number of induced abortions (certainly considerably less according to the Guttmacher and WHO estimates).^8^

That the causes may be split up into specific conditions may not affect the argument significantly. After all, if we suppose that resources should be apportioned relative to the burden of mortality, Ord and Berg still have an argument that the total expenditure on chromosomal anomalies ought to exceed spending on induced abortion when added altogether, and clearly pro-lifers do not expend as many resources on chromosomal anomalies as on induced abortion. That said, there being a wider variety of conditions may affect the efficiency of the research and hence, could justify more spending on abortion: if miscarriages had 140,000,000 different causes, it is plausible that the cost to find a treatment for 50,000,000 spontaneous abortions is likely to be higher than the cost of preventing 50,000,000 induced abortions. So, a significant amount may depend on the extent to which the disparate causes of spontaneous abortion are likely to have treatments derived through similar or the same research. Berg and Ord may have a point that pro-lifers, on the whole, have not even attempted this calculation (difficult though it surely is): they may be right that the pro-life community as a whole has a moderate duty to at least attempt a calculation of this sort before deciding that preventing abortion is most cost-efficient.

However, there is a much more powerful reason why the proportion of deaths caused by genetic anomalies (chromosomal or otherwise) and the careful distinction of these genetic anomalies may be important. The reason is that some genetic anomalies—especially those in question here—may be so radical as to prevent the creation of a human organism or, more plausibly, that changes to certain genetic constitutions may not be identity preserving.

We clearly do not want to say that any chromosomal anomaly prevents the entity constituting an organism. People with Turner Syndrome and Down Syndrome are clearly human beings and moral equals.^9^ What about those with tetraploidy? That seems less clear. I suspect we do not know enough about genetics at this point to be able to judge whether most entities with extreme chromosomal anomalies are human organisms.^10^ My own view is that most or all are. In any case, perhaps even uncertainty should make us err on the side of caution, granting them personhood unless we have clear evidence that they are not organisms. So, this response will not do a great deal.

What seems much more plausible is that large changes in genetic constitution may not be identity-preserving. I have previously defended this view (Miller and Pruss, 2017), noting that even some relatively small genetic changes (such as in Tay–Sachs disease, which can be caused by mutation of a single base pair) may not be identity-preserving. Would I be the same person if I had Tay–Sachs or Turner Syndrome? Or if I had the opposite assortment of sex chromosomes? It is very plausible that I would not be. If so, then it seems like most aneuploidies—and hence, a very large proportion of spontaneous abortions—result in a different human being to the human being they would have formed if the anomaly had not occurred.

If substantial genetic changes are not identity-preserving, then many genetic anomalies are not treatable. This is *not* for technical inability—which could be solved with enough research—but because it is *metaphysically impossible* to treat the genetic anomaly without altering the identity of the individual. Hence, these deaths are unpreventable in a way that cannot be solved by research, and so it is difficult to see how any resources could be devoted to them.

On a certain view—which I suspect most pro-lifers would endorse—this matters a great deal. On this view, it is not necessarily wrong—and certainly not comparable to murder—to bring into existence a genetically anomalous child, even if one could have acted differently and brought into existence a healthy child.^11^ Indeed, intuitively, the main reason why it might be wrong to bring into existence a severely disabled child is the suffering that the child would bear. This reason is nullified in the case of a child who will not suffer because they die before their nervous system is sufficiently developed, or if the particular kind of suffering would only occur at a later point after the spontaneous abortion (e.g., from hypoxia due to inadequate ventilation). In short, it is not comparable to murder to bring into existence a child with, say, trisomy 2, who will die before birth.

This is important because Ord and others suggest that these deaths are preventable by preventing that particular conception, with that genetic constitution, from occurring. But, preventing a person from ever existing is hardly morally equivalent to saving their life, even though both are spared death (in different senses). A person with trisomy 2 is not benefitted by being prevented from ever existing in the first place. Conversely, they are not harmed by being brought into existence for a very short time. Thus, it makes no sense to say that these are preventable deaths, or to say that pro-lifers have a duty to prevent these conceptions taking place. They are preventable deaths only in the sense that they stop someone from ever existing in the first place: by that measure, every death is preventable. Clearly, this is implausible and should not have a significant impact on policy—preventing deaths in the sense of preventing people with a short life expectancy from existing is simply not a duty, certainly not a duty comparable to preventing homicides. At the very least, we can say that bringing into existence persons who will die shortly after their conception, and thereby in some sense causing those persons’ death, is obviously not equivalent to murder. Arguably, it is not wrong at all. Hence, there is no argument for thinking that pro-lifers should seek to prevent these deaths over the deaths caused by induced abortion.^12^

I said earlier that the distinction of specific genetic anomalies may be important for this response. Some genetic changes are plausibly identity-preserving, while others are not, and so pro-lifers may have some reason to research gene therapy for some individuals as a way to save lives. But, that number of individuals will probably be a much smaller number than the number of lives lost to aneuploidy. It is certainly plausible that many aneuploid individuals are literally untreatable—treating them is not even metaphysically possible. And so the number of *preventable* miscarriages is plausibly *much* lower than 140 million.

If I am right that the number of induced abortions is vastly lower than 56 million, these points will still not suffice. For, even if we exclude all those cases of “essential” aneuploidy (and other genetic anomalies which are literally untreatable), the number of spontaneous abortions with preventable causes may well still exceed the number of induced abortions, and so still generate a potential inconsistency for the pro-lifer.

Still, everyone in the debate is agreed that pro-lifers should ensure that *something* is being done to save lives lost in miscarriages. We should also agree that the loss of life due to miscarriage is seriously regrettable—this is far from unintuitive, however. Indeed, it is difficult to give an account of the badness of death that does not have the implication that spontaneous abortion is a very bad thing, not to mention the grief women suffer from miscarriage—enough for the UK’s leading abortion provider to refer to it as “The inconceivable grief of baby loss” (British Pregnancy Advisory Service, 2020).

As it turns out, much is already being done to try and prevent miscarriages: a great deal of research, involving many millions of pounds, is already being done on the causes of miscarriage. Even in 2005, over $2.9 billion was spent globally on genetics research (Pohlhaus and Cook-Deegan, 2008), while the fertility business makes an estimated $25 billion a year in sales, much of which presumably goes into research on helping pregnancies come to term (The Economist, 2019). Sometimes this research is carried out specifically by pro-life or Catholic organizations (see NaProTechnology, 2020). It goes without saying that there is already enormous global expenditure on public health campaigns trying to stop smoking, on research on chronic conditions contributing to miscarriage like diabetes, and so on. Even recurrent miscarriage is the subject of a great deal of research. There is no large research budget for “miscarriage” generally because it has a wide variety of causes. But, for those individual causes, there are enormous research budgets. While a quantitative analysis is beyond the scope of this paper, it seems plausible that money spent on research helping to prevent miscarriages *vastly* exceeds money spent on preventing abortion, even when controlling for the numbers of miscarriages and abortions.^13^ If so, it is hardly clear what pro-lifers are expected to do. We do not expect researchers in non-malignant thyroid disease to spend *any* time, let alone a large majority of it, on researching cancer, despite the far greater mortality of the latter. Why? Because other people are already doing it, disproportionately, even. Likewise, given the relatively meager budgets of pro-life organizations, it is hard to see why they should spend it on miscarriage research, given the vastly greater sums of money already being spent on such research.^14^

## IV. OTHER REASONS FOR PRIORITIZING ANTI-ABORTION ADVOCACY

Before turning to the argument that I wish to develop in detail, it is worth briefly summarizing just a few reasons (in addition to the considerations above) why pro-lifers might reasonably consider abortion to be more worth preventing than miscarriage. These will be of relevance to my central argument, as we shall see. We begin with a series of reasons why abortion is more degrading than miscarriage:

1) Methods of abortion are often degrading. In England and Wales, for example, around 10,000 abortions every year involve dismembering the live fetus, a fetus at around 14 weeks’ gestation or more (Department of Health and Social Care, 2020). In some other countries, surgical abortions (of which those at later gestations involve dismemberment) are more common still (Guttmacher Institute, 2019; Popinchalk and Sedgh, 2019). Public opinion in the U.S. shifted significantly toward the pro-life position in the mid-1990s (Gallup, 2020) around the time partial-birth abortion began to be publicized, eventually leading to a federal prohibition. Partial birth abortion was normally performed on healthy babies in healthy mothers (New York Times, 1997) and involved delivering a living child all except for the head, at which point “blunt scissors” are forced into the baby’s skull so that the “skull contents” (i.e., the brain) can be suctioned out (Haskell, 1992). One nurse testified:

Dr. Haskell went in with forceps and grabbed the baby’s legs and pulled them down into the birth canal. Then he delivered the baby’s body and the arms—everything but the head. The doctor kept the head right inside the uterus … The baby’s little fingers were clasping and unclasping, and his little feet were kicking. Then the doctor stuck the scissors in the back of his head, and the baby’s arms jerked out, like a startle reaction, like a flinch, like a baby does when he thinks he is going to fall. The doctor opened up the scissors, stuck a high-powered suction tube into the opening, and sucked the baby’s brains out. Now the baby went completely limp … He cut the umbilical cord and delivered the placenta. He threw the baby in a pan, along with the placenta and the instruments he had just used. (Supreme Court of the United States, *Gonzales v. Carhart*, 550 U.S. 124 (2007), 8)

In my experience, those hearing this description have described it as straightforward murder, if not a crime against humanity, *regardless* of their general position on abortion. In any case, it is not difficult to see why someone would regard this, other things being equal, as worse than a miscarriage, even than a stillbirth of the same gestation. It is worth noting that recent research suggests fetal pain from 12 weeks is distinctly possible—and certainly at later stages (Derbyshire and Bockmann, 2020).
A ban on this procedure was vetoed by President Clinton, opposed by the majority of House and Senate Democrats, and even declared unconstitutional by four Supreme Court judges.^15^ Although partial birth abortions constituted only a small proportion of abortions, it was responsible for an estimated 3,000–5,000 abortions a year, 500–750 of which were after 7 months’ gestation (Johnston, 2007). By contrast, Presidential candidate Elizabeth Warren (2019) described children dying by gun violence in the US as a “national health emergency,” responsible for around the same number of deaths (Cunningham et al., 2018). Partial birth abortions alone counted only for a very small proportion of surgical abortions in the US. This is relevant because a major political party in the most powerful country in the world supporting this kind of degradation—while claiming that the same number of deaths from another cause is a “national health emergency”—adds a further level of systemic institutional degradation against human beings in the womb.
2) Abortion, on the pro-life view, involves a violent attack within the family—hence, many early Christian writers referred to it as “parricide.” As well as parricide being particularly destructive within a family, widespread parricide plausibly results in a more general destruction of the family by the normalization of the dispensability of children and the erosion of the appreciation for motherhood. Since, according to the Universal Declaration of Human Rights Article 16, the family is the “natural and fundamental group unit of society and is entitled to protection by society and the State,”^16^ the widespread breakdown of the family is plausibly a unique and serious evil to be prevented at any cost, in contrast to miscarriage which—while causing families much grief—does not involve the same kind of relational fracturing. Relatedly, abortion contributes to a widespread culture of the dispensability of children, which plausibly contributes in the same way to more general societal harms. Along similar lines, pro-lifers often think that abortion contributes to a sexual culture that is likewise destructive to human flourishing (and disproportionately so for women and the poor) by severing the link between sex and procreation (Alvaré, 2011).
3) Abortion involves the systematic and state-sponsored violence against a particular class of people, which most of us would regard as worse than the natural death of the same number of people. One reason we are appalled at the Holocaust, the Rwandan genocide, the Armenian genocide, and so on, is because of the systematic and state-sponsored violence specifically impacting (and even targeted toward) a vulnerable demographic group on a large scale. There are multiple elements here, all of which no doubt contribute to the horror of these genocides, and all of which seem on initial inspection to justify greater attention than the equivalent number of deaths by natural causes. Even on much smaller scales, many of us think that it is reasonable to take radical action over violence against vulnerable groups, exceeding our response to natural deaths among those groups. Perhaps the most salient example is from the Black Lives Matter movement. The Guardian reports that in 2016, 39 unarmed black men were killed by US police. Compared to other causes of death, this is a relatively small number. Yet, we rightly realize that it is much more salient than 39 deaths from natural causes, justifying a much more significant public response. As Colgrove puts it, “BLM has a particular target. It is not solely fixated on saving as many lives as possible. It is aimed at revealing (and undoing) systematic injustice that is directed toward certain people” (Colgrove, 2021).
4) Abortion involves the systematic *dehumanization* of a class of people. One of the most basic rights of human beings is to be recognized as a person,^17^ and it seems clear that the dehumanization of certain classes of people (whether Jews, black slaves, Tutsis, disabled people, or others) is a serious aggravating factor to many crimes, especially genocides. This raises a particularly salient point: as well as dehumanization being an aggravating element of mass killing, working to oppose dehumanization constitutes the bulk of pro-life work in many countries. It may even be that humanizing the fetus is the best way to garner public support for putting resources into preventing miscarriage, and that discussion of abortion is one of the best ways to demonstrate the humanity of the fetus (the Partial Birth Abortion Ban Act and its effect on public opinion suggest this may be the case).^18^
5) In the case of abortion, tens to hundreds of millions of dollars are spent advocating *for* abortion all over the world as a basic human right and as a part of essential health care. There is not a similar movement promoting miscarriage. This means that the dehumanization involved in abortion is not only ideological: it is institutionalized in our economic system, with hundreds of millions of dollars invested into its propagation.^19^
6) In the case of abortion, the killing and dehumanization receive the imprimatur of the state and the law (and institutions like the UN and the WHO, insofar as they are able to make pronouncements not reflecting international consensus). The expressive function of the law is powerful—we can see this by asking whether we would allow, for example, the state to declare that African Americans are not fully human, if for some reason it meant that they received better treatment.^20^ People are divided on this question—which is enough, I think, to show that the expressive function of the law *plausibly* might outweigh a certain number of lost lives. It is one degradation to have a large number of the population dehumanize you: it is another level of degradation to have this enshrined in law, and in the major political institutions. Thus, the ideological and economic weight behind abortion is compounded by political weight; it is plausibly more degrading—and certainly more of a problem—for the dehumanization of a class of human beings to have the imprimatur of the ruling institutions.^21^
7) If, as many prominent philosophers have thought, virtue is a *constituent* of well-being, then on the pro-life view abortion is also extremely harmful to those participating in abortion. More generally, if you think that our character is more important than our experiences, you would have a strong interest in preventing abortion that does not apply to miscarriage. There is also evidence linking abortion with increased suicide rates and increased mortality (Fergusson et al., 2013; Karalis et al., 2017), suggesting more lives may be lost due to abortion (though of course, not enough to match those lost in miscarriage). While this does not necessarily make abortion more degrading for the primary victim, it is another reason to oppose more strongly than miscarriage.^22^
8) One of the primary obstacles to generating substantial societal support for miscarriage and abortion prevention is the view that unborn human beings are not really human, or are not equally morally considerable. (Effective) anti-abortion advocacy helps to remove this obstacle for both causes, and perhaps more effectively than miscarriage prevention advocacy.

As a sense of what is at work here, imagine tens or hundreds of thousands of members of another vulnerable group were dismembered alive each year, with the social, legislative, and financial support of the most powerful governments around the world, as well as immensely powerful NGOs, the UN, and the WHO. Imagine this was just the tip of the iceberg for many millions more who were killed in less graphically violent ways. Now imagine there was already a great deal of research being put into saving these people from dying due to natural causes. It seems overwhelmingly likely to me that we would think we had a mandate to invest significantly more resources to stop the state-sponsored killing, compared to preventing the equivalent number of people dying from natural causes. We certainly could not rightly criticize human rights groups for paying significantly more attention to the former. The same situation is broadly what we have in the case of abortion, from the pro-life viewpoint. Indeed, comparisons to mass killings such as the Holocaust are perhaps the best way to understand the pro-life mindset *for our purposes here*, since such comparisons are the intuitive reason many pro-lifers offer for making abortion a central political and social priority.

I make no claims here about exactly to what extent the large numbers involved contribute to the horror of mass killings and systemic dehumanization. It might be that what made other mass killings so abhorrent is the large numbers. If so, then this only serves to make abortion more serious. In the case of abortion, we appear to have both factors: systemic violence and degradation, as well as enormous numbers.

Since my central argument is to do with the *respect* owed to victims of killing, all of the considerations here which pertain to the *degradation* and *dehumanization* involved in abortion will amplify the force of that argument, to which we now turn.

## V. THE CENTRAL ARGUMENT

The suggestion that killing and letting die are morally inequivalent is hardly a new one, nor is its application in this context. Indeed, it is the obvious response to the argument: it is the most obvious difference between induced abortion and miscarriage. But perhaps it is undermotivated or ad hoc. Why think that they are morally distinct? Indeed, some philosophers have suggested that they are not distinct when all other things are equal (Simkulet, 2019). Here, I propose an argument for thinking that they are distinct, and that this has significant implications for prioritizing the prevention of killing vis-à-vis the prevention of natural deaths.

My starting point is with the badness of death and the wrongness of killing. In the context of abortion, this has been heavily influenced by Marquis’ (1989) seminal work arguing that abortion (and killing more generally) are wrong because they deprive someone of a future life of value. This is a kind of *deprivationist* account of the wrongness of killing, and for many people it is an intuitive account of the wrongness of killing. Marquis’ paper is no doubt popular because it was one of the first major papers to be published for the pro-life position in the recent renaissance of pro-life philosophy. It is also an extremely simple and intuitive argument. As it happens, I think that the paper is largely correct, accounting for one reason why abortion is wrong. Marquis’ work deserves the attention it has received, including in recent discussions.^23^

Now I do not, in fact, think that it is the central reason for the wrongness of abortion. To help explain my position, consider another feature of Marquis’ argument: it implies that the wrongness of killing is closely tied to the badness of death, which is widely variable. As Blackshaw and Rodger (2019) note, the badness of death differs a large amount between persons. But, if killing is wrong primarily because it deprives someone of a future life of value, then the wrongness of killing can vary from extremely wrong (in the case of embryos) to minimally, or perhaps not wrong at all (in the case of people killed near the end of their lives, or in the case of severely disabled people who are not able to enjoy much). The precise results in these cases depend on what is considered “valuable” about life, but it seems as though defenders of Marquis tend primarily to emphasize valuable experiences, rather than the inherent value of life itself.

It has been claimed that this kind of deprivationism is the standard view among pro-lifers. Indeed, Simkulet criticizes Friberg-Fernros’ defence of the significance of the distinction between killing and letting die by saying that “this view is at odds with the commonsense antiabortion position, grounding the wrongness of induced abortion not in the death of the fetus, but in the act of killing (or in disconnect cases, letting die)” (Simkulet, 2019).

In fact, however, there are many reasons to think that the orthodox pro-life position is not based solely (or even primarily) on the badness of death. I would go further: it is actually intrinsic to the pro-life account that the wrongness of killing is not correlated with the badness of death. For example, pro-lifers are typically just as fiercely opposed to euthanasia or assisted suicide—even if the patient has almost no life of value remaining—as they are to abortion, which prevents many decades of valuable life. If killing is wrong because it deprives the victim of a future life of value, abortion should be considered orders of magnitude (if someone is killed by involuntary euthanasia a day before they were likely to die anyway, then abortion would be roughly 365 × 70 = roughly 25,000 times worse, or more if the euthanasia recipient is looking forward only to a day of misery) more wrong than euthanasia. But, pro-lifers tend to consider them roughly commensurate—or, at the very least, not as though one is 25,000 times more serious than the other. Pro-lifers object vehemently to the killing of people who are not likely to enjoy significant subjective goods in the future—for example, anencephalic children in the case of abortion,^24^ or severely disabled or very elderly/terminally ill people in the case of euthanasia. The enormous effort to prevent the killing^25^ of Terri Schiavo and the more recent case of Vincent Lambert are a testament to the seriousness with which pro-lifers take killing even in such cases. This is strong evidence that pro-lifers are not primarily pro-life because of potential future subjective goods to be experienced by the victim.

Second, the “traditional” pro-life approach was to speak of the “sanctity” of life. This terminology fits very well with the sense of intrinsic value and respect in the account I describe, and not so well with the view that life is valuable because of the subjective goods that may be experienced. Third, it is a staple of the pro-life view that life is intrinsically valuable—this is at odds with a view that takes life to be valuable primarily because of experienced goods. Pro-lifers make an extremely large point of valuing all human life, including where the quality of life may be very low. Pro-lifers consistently focus on the value of those with severe disabilities, which is surprising if they think the value of life is primarily determined by experienced goods. Fourth, many pro-lifers are pro-life for religious reasons, and religious prohibitions on killing have rarely, if ever, spoken of future goods. On the contrary, the Christian tradition, at least, has expressly used terminology more suitable to the respect theory I describe. This is perhaps best exemplified by Lactantius’ comments on killing at the turn of the fourth century: “Therefore, with regard to this precept of God, there ought to be no exception at all; but that it is always unlawful to put to death a man, whom God willed to be a sacred animal” (Divine Institutes, 6.20). Finally, as Friberg-Fernros (2019) points out, the intention is extremely important for the pro-life view, which is why pro-lifers allow the *foreseen* death of fetuses to save the mother’s life, without ever allowing the intended death of fetuses.

Pro-lifers probably come across as being deprivationists primarily because many of them hold that deprivation is an additional reason why abortion is wrong, and—within philosophy—because of the influence of Marquis’ article on the debate in recent decades. But, that is no reason to suppose that the majority of pro-lifers *only* or primarily rely on deprivationist arguments for their view on abortion.

What all this suggests is that pro-lifers do not think that the wrongness of killing and the badness of death are very closely related—at least, not if the “badness of death” is measured in terms of the deprivation of positive subjective experiences.

This is important for the following reason: the comparison between spontaneous abortion and induced abortion relies crucially on one specific similarity: they both involve the same deprivation to the individual. Both individuals lose the large majority of their lives, and this is supposed to be what makes spontaneous abortion as pressing for the pro-lifer as induced abortion (and more so, once the numbers are added). But, if the wrongness of killing is not very closely tied to the badness of death, then the fact that both are similarly deprived (and hence the badness of death is roughly the same for each) may be of negligible relevance. Simply put, my argument runs thus:

1. The main similarity between babies or fetuses killed by abortion and those whose lives are ended in miscarriage is that they are both deprived of the same subjective goods.^26^
2. However, deprivation of subjective goods is not the primary determinant of the wrongness of killing.
3. If 1 and 2, then the main similarity between abortion and miscarriage has little to do with the wrongness of killing.
4. Hence, the main similarity between abortion and miscarriage has little to do with the wrongness of killing.

If this is right, then drawing attention to this similarity between abortion and miscarriage does little to persuade the pro-lifer that they should give both equal attention. But, it would help if we had (a) some motivation for premise 2 and (b) an alternative account of the wrongness of killing that pointed toward (even if not explicating precisely) the reason we may be more worried about killing by abortion than death by miscarriage.

McMahan (2002), in his magisterial volume on the ethics of killing, offers pointers toward both of these.

## VI. IS THE DEPRIVATION ACCOUNT TRUE?

McMahan calls this sort of account the Harm-Based Account—killing is wrong because of the harm inflicted on the individual, harm being conceived as the loss of future goods. McMahan thinks that a fatal flaw to this account is that it thinks identity is what matters. In fact, according to McMahan, it does not: what matters is prudential unity relations—psychological relations tying an individual’s psychological life together. Hence, McMahan talks of “time-relative interests,” where these are essentially ordinary interests, whose importance is augmented or discounted, depending on the strength of the psychological connections between an individual at different times.^27^

McMahan goes on to suggest that the Time-Relative Interest Account of the wrongness of killing is likewise deficient, however, at least for persons. Why is this? McMahan says that both the Harm-Based Account and the Time-Relative Interest Account have the implication that killing can be more or less wrong *depending on the quality of the victim’s life*.^28^ This, McMahan says,

profoundly offends our sense of the moral equality of persons…The common view, in short, is that the wrongness of killing persons does not vary with such factors such as the degree of harm caused to the victim, the age, intelligence, temperament, or social circumstances of the victim, whether the victim is well liked or generally despised, and so on. (2002, 234–5)

McMahan dubs this the Equal Wrongness Thesis.

The Equal Wrongness Thesis is intuitively plausible. Human equality, and its relevance to fundamental inviolable rights, are popular ethical ideas, on which it seems reasonable to base ethical opinion in the absence of countervailing considerations. Note that if it is true, then premise 2 of the above argument is true: if all killings are in some sense equally wrong,^29^ specifically if they are equally wrong regardless of the quality of the anticipated goods in an individual’s life, then the wrongness of killing cannot be dependent on the deprivation of one’s future. Pro-lifers do not need to prove the Equal Wrongness Thesis to respond to the arguments of Ord et al.—they just need to show that there is a plausible reason for their discrepant approaches toward abortion and miscarriage. The Equal Wrongness Thesis is certainly at least plausible.

## VII. IS THERE AN ALTERNATIVE ACCOUNT OF THE WRONGNESS OF KILLING?

McMahan goes on to suggest an alternative account that can explain the pro-lifer’s discrepant attitudes, along with a number of other ethical difficulties. It is, therefore, a fruitful theory, and one that I believe accords very well with our intuitions. McMahan suggests the following:

If the killing of persons is always equally wrong, and if all persons are of equal worth, the wrongness of killing may be a function of the worth of the person (rather than of the value of the person’s subsequent life)…a person, a being of incalculable worth, demands the highest respect. To kill a person…is an egregious failure of respect for the person and his worth. It is to annihilate that which is irreplaceable, to show contempt for that which demands reverence…Killing is, in short, an offence against what might be called a *requirement of respect* for persons and their worth. (2002, 242)^30^

McMahan eventually settles on a Two-Tiered Account, according to which the killing of *persons* is wrong for these sorts of reasons, but for those below the threshold of personhood and the threshold of respect, the wrongness of killing is governed by the Time-Relative Interest Account.

Let us give a few clarifications on this account. First, on this account, “the worth of the victim is entirely independent of the value…of the contents of his possible life in the future” (2002, 243). Hence, it supports premise 2 of my argument. Second, it supports the Equal Wrongness Thesis, since it says that all killings of persons are, in a basic sense, equally wrong.

Third, it does not say that, *all things considered,* all killings of persons are equally morally wrong, despite appearances. McMahan explains this more carefully: “Despite my choice of label, the Equal Wrongness Thesis does not imply that the wrongness of killing persons never varies. It is compatible with that thesis to recognize that the wrongness of killing can vary in ways that are consistent with the fundamental moral equality of persons” (2002, 235). He goes on to describe a number of ways in which the wrongness of killing can vary: more people killed, worse motivation, intention rather than foresight, and so on. The crucial point of equality is, as I wrote above, that it does not vary according to the worth of the victim (since all victims are equally valuable) or the worth of the victim’s life (since this is irrelevant, on this account).

McMahan defends the respect account of the wrongness of killing at greater length, but let me add some additional motivations for this thesis:

First, as described, it is an account that best fits our intuitions about the fundamental moral equality of human beings, and the connection between the equality of human beings and their most basic rights.

Second, it explains why the right to life is inviolable, as opposed to being potentially expendable, depending on the potential benefits. The International Covenant on Civil and Political Rights^31^ says that the right to life is not violable, even in time of public emergency threatening the life of the nation, for example. This, of course, fits with our intuitions regarding the impermissibility of killing, even if it were possible to save more lives (as with the innocent healthy person who is killed to save five lives with organ transplants).^32^ Those of us who are opposed to capital punishment and torture (among other things) may find the generation of inviolable rights on the basis of *respect* especially intuitive as well. It connects these rights in a plausible way with the *absolute* or *infinite* worth of the individual.

Zylberman (2016) has a helpful discussion of the concept of human dignity and its connection to these rights. Zylberman first quotes Kant: “In the kingdom of ends everything has either a price or a dignity. What has a price can be replaced by something else as its equivalent; what on the other hand is raised above all price and therefore admits of no equivalent has a dignity” (Zylberman, 2016, 204).

Zylberman then describes some implications of this view: in particular, that human dignity can never be traded away, even for something else with dignity, and hence results in an absolute prohibition on certain kinds of conduct, such as torture. This means it can also explain the equal wrongness of killing. The respect account can plausibly claim that human life is priceless, or infinitely valuable, and hence that all human beings are equally valuable. If the wrongness of killing is tied to their value, it can explain the equal wrongness of killing.

Third, it takes seriously our special status as *persons* rather than just sentient beings, connecting this in a plausible way to our inviolable right to life.

Fourth, it has strong historical precedent (and therefore the intuitive support from the “democracy of the dead,” as Chesterton put it): the U.S. Declaration of Independence ties together our equality and the unalienable right to life,^33^ and the Universal Declaration of Human Rights, in its opening line, reminds us that “recognition of the inherent dignity and of the equal and inalienable rights of all members of the human family is the foundation of freedom, justice and peace in the world.” Article 7 gives: “All are equal before the law and are entitled without any discrimination to equal protection of the law.” Arguably, liberal democracy is founded on a concept like respect, along with its moral implications. As we saw earlier, the Christian tradition on which our conception of rights and equality is historically grounded (Spencer, 2016; Holland, 2019) has generally emphasized the “sacredness”—the inviolability due to respect—of human life.^34^

Fifth, it makes a great deal of sense to many wrong actions which do not seem to harm anyone in a way that is appreciable to the victim. There are many things which are wrong but from which no one *necessarily* consciously suffers as a result.^35^ For example, desecrating someone’s grave, deliberately contradicting or desecrating your loving, caring mother’s dying wish, privately laughing at another’s misfortune, cheating on one’s partner, secretly putting pork in a devout Muslim’s meal, watching child pornography, cannibalism, necrophilia, consenting to sell oneself into slavery, and so on.^36^ That these wrongs *disrespect* the victim rather than “harming” them seems the best explanation of their wrongness.^37^

Sixth, it explains our intuitions about particularly aggravated killings: for example, racially motivated or particularly degrading killings.

Finally, it fits very naturally with the pro-life view, which in its standard form says that the right to life is inviolable. Since the allegation is that the pro-life view has an inconsistency, this is a helpful virtue for the pro-lifer.

## VIII. DIFFERING NORMS FOR KILLING AND LETTING DIE

This account of killing has a lot to commend it. Now how does it affect our central question? In this next section, I suggest that as a result of this account of the wrongness of killing, there are different norms for killing and letting die.

Here, I largely set aside the question of the extent to which intention to harm is important. To go into the literature on intention would take us too far afield, but it is certainly possible that the intention to harm is part of what makes killing necessarily disrespectful. In that sense, intention may well be a substantial part of my argument here. If so, my argument would neatly integrate and explain why intention is so important to the distinction. If not, my argument still works.

All I add here, therefore, is to say that it is deeply intuitive that intention makes an enormous difference to the morality of an action. To take a very simple example: it is permissible to perform actions foreseeing the death of civilians in wartime, so long as the action taken fulfills certain other strict criteria. By contrast, intentionally targeting civilians is a war crime. Foreseeing the death of a person when adjusting a policy to save five others is OK. By contrast, killing someone to use their organs for transplants is not. Examples could be multiplied endlessly, but the intuition is strong. Since, as we see, the intention in performing an abortion and in allowing a miscarriage is (usually) very different, there is an obvious reason to treat them discrepantly. What I propose is that the respect theory of the wrongness of killing may channel the role of intuition in these cases—intentionally killing a fetus is inherently disrespectful, while foreseeing the death of the fetus is not necessarily disrespectful.

The argument at this point is, again, very simple. Killing is inherently disrespectful—a violation of human dignity. It is impossible to (intentionally) kill in a way that respects the value of the victim’s life, and hence there is an absolute prohibition on it.^38^ Now failing to save is not *inherently* or *necessarily* disrespectful. There are many reasons why one might fail to save someone that do not necessarily represent a failure of respect for that individual.^39^ Hence, there is no absolute prohibition on failing to save. Saving someone from natural death often is a duty for various reasons—indeed, sometimes the failure to do so is a failure of respect. But, it is not necessarily so. Hence, different norms apply to each: killing is always wrong, for the reason that it is necessarily a failure of respect. Failing to save may or may not be wrong, depending on the details of the individual case.

At this point, we can respond to an objection that has been raised by a number of writers. Simkulet, for example, argues that once scenarios involving killing and letting die are made similar in every other respect, there is no moral difference between the two. In response, note that *even if true*, this does nothing to damage my argument. For one of the relevant factors that need to be made similar for the two scenarios to be comparable is *a failure of respect*. Killing, I have said, necessarily involves this. Failing to save only contingently does so. Where failing to save does constitute the same kind of failure of respect, it may well be just as wrong as killing.^40^ But, in cases where failing to save is not borne out of a failure of respect, it is not necessarily as wrong. This result is all that is needed for my overall argument to succeed. Where failing to save is not a failure of respect, clearly there are other norms that govern such situations—and these norms may be significantly different from the norms governing killing.^41^

Let us motivate the idea that the respect account generates differing norms a little more. First, I highlighted earlier how many of the bad features of abortion emphasized by pro-lifers are bad specifically because they are *degrading*, or because they disrupt a respectable part of the natural order (such as the love between members of a family). This adds more weight to the idea that killing (particularly in abortion) involves a lack of respect, while spontaneous abortion lacks those same features.

Second, the general reason we fail to save people is clearly not a lack of respect; it is a lack of resources. Indeed, it is *impossible* to save everyone. This creates a clear asymmetry with killing: it is entirely possible never intentionally to kill anyone, yet it is completely impossible to save everyone. If it is impossible to save everyone, then it is wildly implausible to claim that failing to save someone necessarily involves a failure of respect.

Third, the infinite value of human life easily translates into a principle on killing: do not kill. What is the equivalent principle of saving life? Because it is not possible to save everyone, it is difficult to see what this could be. Certainly, it is difficult to see what plausible principle this could involve. Put another way: it is possible for us to achieve infinite utility in the realm of killing: simply by not killing at all. But, it is harder to understand what infinite utility would look like when it comes to saving lives.

Fourth, further intuition supports different norms for the two cases. We would potentially kill in order to prevent a murder or genocide. This seems to be broadly the only situation killing would gain widespread support.^42^ However, we would not at all countenance the thought of killing one person to save more people from another disease (as in the transplant case). This suggests that there are different norms for the two situations.

Fifth, it is almost universally agreed that killing and letting die are morally different. This is clear in Anglo-American law and international law, among many other (perhaps all) jurisprudential systems. Hence, there are many reasons to think that the norms governing each situation are distinct.

What norms could govern failing to save? I do not aim to set out a comprehensive algorithm: just to list some of the possibilities and their plausibility. First, of course, a failure to save can be a failure of respect, which would make that failure prohibited. Aside from this, we could be guided by many considerations:

- How much such persons might be able to contribute to the rest of society (e.g., if they have the cure for cancer).
- How many expected years of life that person has remaining.
- How many vulnerable people that person has depending on them (including if they are pregnant).
- Whether that person belongs to a protected class of human beings disproportionately affected by systemic injustice.
- How likely the person is to survive the intervention.
- Prudential unity considerations (i.e., the time-relative interest account).
- Whether that person would prefer to live or die.
- Other competing goods—not just saving lives, but other cultural goods and other sources of human flourishing, and the prevention of harm and/or misery.
- The proximity of the person.
- Special relationships we might have with the person.
- The circumstances and cause of death: including whether the person is being killed by another person or by natural disease, and the reason for their being killed (if homicide).

Most of us already believe that at least some of these are relevant to whether we have a duty to save someone’s life, and if so, which lives we should save (given limited resources). So, our intuitions already support this sort of reasoning—*even though* most of us agree that humans are equal and have equal rights. This supports the idea that an equal right to life is primarily a negative right, as described. Most people endorse an account like this: killing is equally wrong across all persons, but failing to save can be right or wrong depending on a wide variety of factors. As some examples of the above: most people agree that you should save someone who has a cure for cancer over someone who does not; the idea of spending limited resources to preserve as many *years* of life as possible (hence discriminating between persons) is not only widely accepted but built into the foundations of health economics and resource distribution; most people think you have a duty to save your child over someone else’s child, and so on.

We even tacitly grant that competing goods that can justify not saving someone’s life include goods that do not save the life of another person. Almost every country in the world, and almost every person in the developed world, at least, spends a large amount of money or other resources on things that do not save lives, when they could have contributed toward saving a life.^43^ At the same time, we would still all grant that killing people in the developing world to make money for those same goods is clearly morally monstrous.

Almost everyone endorses policies that result in more deaths because they think that when it comes to *saving* lives, this can be weighed against other competing goods. As an example related to abortion: most people support delayed childbearing in order to improve equality between men and women, even though the subsequent delayed childbearing leads to increased maternal mortality (Koch, 2012).^44^

Likewise, it is uncontroversial that a central part of medicine is not only the preservation of life but also the alleviation of misery. Those in health care realize they could save more lives by shifting all the money from misery prevention into life preservation; almost no one thinks we should do so.

All that is needed for my argument is the theoretical result that some of these considerations can outweigh numbers alone. If it is permissible to save one young child over two 90-year-olds with terminal illnesses, then we have granted that equality and the equal wrongness of killing do not mean that we cannot discriminate on any other basis when it comes to saving lives. And, that is enough to generate the possibility that preventing abortion or curing cancer ought to be a bigger priority than preventing miscarriages. If it may be permissible to stop an ethnic genocide against 10,000 people over a disease killing 10,001, the principle is proven.

To use an example that may be more (though far from entirely) analogous to abortion, suppose a disease has been unleashed, and you can save only:

1) A group of ten young girls, deeply integrated into their local communities, whose death would be extremely painful and degrading, and would lead to much misery for both the victims and perennially for their families.
OR
2) A disparate collection of eleven hermits with no social contact at all, who would be missed by no one, who are in comas, and who would die quickly and painlessly.

I am not going to adjudicate in this scenario; I do not need to. All I need is to show that the answer is not *obvious*, even for those of us completely committed to human equality. We would all be agreed that killing the hermits is absolutely wrong, no matter the benefit. But, we might reasonably think that allowing a greater number of hermits to die to save the tightly bonded community is permissible. And that is enough to show that numbers are not the only consideration when it comes to saving lives.

We have, then, a plausible theoretical explanation for why different norms governing the different situations apply. We also have a wide range of intuitions regarding practices mostly already adopted, that suggest we can discriminate when it comes to saving lives. We also firmly believe that this is compatible with our deep commitment to human equality. The view that different norms govern killing and letting die appears to have robust justification.

We are now in a position to respond to Ord’s pre-emption of this strategy. Ord says that the difference between killing and letting die does not matter; he can sidestep this question by directly comparing miscarriage to other natural conditions leading to many deaths, such as cancer. As we can now see, this move does not work. Not only does the difference between killing and letting die justify saving fewer lives from killing than from natural causes, a close examination of the wrongness of killing and the wrongness of failing to save reveals very different norms governing each case. Saving lives involves asking many questions other than simply: “how many lives?” Indeed, there are compelling arguments to show that sometimes saving fewer lives is reasonable. With this established in principle, it is not clear what argument Ord can make. He has to show not only that spontaneous abortion kills a large number of people, but that no reasonable overall assessment could justify spending more on other causes. That argument looks to be very difficult to make.

Essentially, there are two different questions. Why prioritize the prevention of abortion over the prevention of miscarriage? For all the reasons I have given throughout this paper. Ord suggests there is a second question: why prioritize the prevention of cancer, coronavirus, and so on, over the prevention of miscarriage? The answer is because the norms for killing and saving are different, and the norms for saving do not require treating every life saved as equivalent, nor do they require treating every life saved as being of overriding importance to other social concerns (such as the prevention of misery, the preservation of community, and so on). We are able to preserve the equality of human beings and the equal wrongness of killing, at the same time as triaging when it comes to saving lives on the basis of a wide range of other considerations.

We can also now respond to Simkulet’s (2019) allegation that pro-life responses appealing to discriminating factors between lives can justify abortion. This would perhaps be the case if abortion were merely a failure to save. However, since abortion is (at least typically) killing,^45^ Simkulet’s response fails.

It is worth briefly responding to the anticipated objection that when it comes to pro-life lobbying, *all* work is rescuing, rather than refraining from killing. Pro-lifers are not just refraining from killing; they are rescuing people, even if from killing rather than from disease or misfortune. This is true, and it is important: after all, it follows that pro-lifers cannot save *everyone* from being killed, and for the reasons explained here they therefore have no infinitely strong obligation (as they do against murder) to try and save children from abortion. Failing to advocate against abortion is not necessarily a failure of respect (though it can be). However, this consideration does not impugn my argument. I have not argued that pro-lifers must advocate against abortion at the expense of anything else or that failing to do so necessarily results from a lack of respect tantamount to killing. Rather, I have claimed and argued that the reasons for saving lives from abortion are significantly stronger than the reasons for saving lives from miscarriage, and hence can justify a greater focus on the former. I make no argument here for how strong those reasons are in absolute terms, and whether they suffice to make abortion a pre-eminent social and political priority.^46^

All that is needed for my argument to work, therefore, are the following claims:

1) The norms for killing and letting die are distinct.
2) Norms other than the equality and inviolability of persons (and numbers) are applicable in the case of letting die.
3) Some of these norms in the case of letting die can outweigh numerical considerations.

I submit that each of these is not only plausible, but widely believed. In this paper, I have sought to elucidate some of the theoretical and intuitive justification behind them. It might be that Ord, Berg, and Simkulet believe that pro-life efforts are still disproportionate when all these norms are taken into account: but that requires more than just a statement of the numbers involved. It takes even more to show that the typical pro-lifer’s priorities are *unjustifiable*. I suspect that this cannot be shown.

## IX. IMPLICATIONS

As I have shown, this account of the wrongness of killing has significant implications for our moral interpretation of killing and failing to rescue, and hence for our prioritization of preventing killing and preventing natural death. The consequences of this are many, and not only limited to the abortion debate. Within the abortion debate, it gives the pro-lifer powerful resources for explaining why they prioritize the prevention of abortion over the prevention of miscarriage. It could also give powerful resources for explaining why a pro-lifer might save a young child over multiple frozen embryos in a “burning building” scenario.^47^ It could help to explain part of the reason killing is much more morally serious than letting die, and hence, it could reinforce a significant disanalogy between abortion and Thomson’s violinist scenario.^48^ It could explain the wrongness of abortion in cases of fatal fetal anomaly, where the child is not expected to enjoy significant positive conscious experience for a long period of time, and perhaps also the wrongness of euthanasia in general. Finally, it could explain the wrongness of early abortion, which many find counterintuitive. As we have seen, the respect account explains the wrongness of certain actions whose victim is never cognisant of the action or its consequences (such as using child pornography). If actions can be seriously wrong—as a violation of respect—without the victim ever experiencing that harm, then perhaps the primary objection to the plausibility of the pro-life view is dispatched, namely, that it is not plausible that a completely unconscious being is worthy of serious moral consideration.

More generally, this account of the wrongness of killing could be part of the reason why we almost universally treat killing as more serious than letting die *and take more measures to rescue people from killing than from natural disease or misfortune*. Since this distinction is a central element of much Anglo-American (and other) jurisprudence, it has considerable importance. The distinction between killing versus letting die is also, of course, relevant for all sorts of ethical debates, and does not need to be explained in detail here. The account can also assist us in prioritizing the prevention of deaths by killing and by natural causes. The distinction between killing and letting die remains respectable.

## Footnotes

1 This appears to have been first published by Annas (1989), who cites Leonard Glantz as the inspiration.

2 Berg and Ord seem to suggest this; Simkulet, by contrast, insists that pro-lifers must be unaware or simply morally monstrous.

3
Condic (2011) offers a fuller discussion.

4 See also Miller (2021).

5 Around 9,000,000 are reported each year in China alone, for example—though for obvious reasons China is likely to have a higher abortion rate than average. See Johnston (2020).

6 As opposed to “euploidy,” where the normal number of chromosomes is present.

7 These proportions may differ by country, depending on the extent to which malnutrition and poorer control of chronic conditions and infections may cause miscarriage. So it may be that—given that these estimates come largely from the developed world—the proportion due to aneuploidy *globally* is actually less than for induced abortion.

8 Even this is a bit simplistic: dying with a chromosomal anomaly does not mean dying *from* the anomaly. After all, some people with Turner Syndrome live long lives: it by no means entails intrauterine death.

9 At least, on the pro-life view. That they are moral equals is highly debatable on the common alternative view that our moral worth is grounded in our psychological capacities. See Miller (Forthcoming).

10 I specify *human* organisms since it is even possible that genetic changes may be so radical as to constitute a separate species—after all, the genetic makeup of different species is largely quite similar. That said, I think it is plausible that the overwhelming majority of cases in question are human, and in many cases constitute organisms.

11 I say “necessarily,” because it may be wrong in some cases: if the intention is to cause suffering, for example. But, if there is a valid reason—for example, wanting to delay childbearing until a point that having a disabled child is very likely but not intended—then it is not inherently wrong.

12 Incidentally, it is also a reason pro-lifers have no significant duty to take particular measures to avoid genetic anomalies recommended by some authors, such as avoiding the rhythm method (which may—speculatively—lead to older and more genetically vulnerable gametes conceiving) or using sperm sorting. Bovens (2006) makes the former argument. Likewise, changing the timing of pregnancy is probably unhelpful from the perspective of preventing deaths, since changing the timing of pregnancy will likely result in a different child.

13 See the figure cited for genetics research here, and Colgrove’s (2021) estimate for money spent on fertility research.

14
Colgrove (2021) makes similar points, while Marino (2008) presents a summary of current research.

15 “The notion that the Partial-Birth Abortion Ban Act furthers any legitimate governmental interest is, quite simply, irrational” (Supreme Court of the United States, *Gonzales v. Carhart*, 550 U.S. 124 (2007), Ginsburg, J., dissenting, 24).

16 See also the International Covenant on Civil and Political Rights, Article 23.

17 See UDHR, Article 6; ICCPR, Article 16.

18
Colgrove (2021) makes this point independently.

19 One might also think that the money spent lobbying for abortion justifies higher spending on abortion insofar as more money is needed to create a “level playing field.” Intuitively, the more money invested in the transatlantic slave trade, the greater the necessity to spend money and resources fighting against it.

20 Advocates of the transatlantic slave trade claimed that keeping the trade legal was the best way to keep it “safe”—banning it would only lead to poorer conditions on the now unregulated ships. See Hague (2008).

21 The same applies from the fifth point, namely, that more resources are needed to create a level playing field regardless of whether the political impetus behind abortion makes it more degrading.

22 A reviewer points out that apathy toward miscarriage is also a moral wrong and could be seen the same way. But the pro-lifer under attack by Ord et al. need not be apathetic to miscarriage. They could think miscarriage is a bad thing and spend some resources trying to prevent it, while spending more opposing abortion.

23 For example, Christensen (2018) and subsequent correspondence.

24 A baby with anencephaly is not able to experience much value—she cannot experience higher pleasures than many animals, and experiences them for a much shorter time. And yet pro-lifers generally consider abortion of anencephalic children to be a very morally serious violation of their right to life, even if much more understandable and exculpable than abortion for, say, career reasons.

25 In the view of many pro-lifers, which is the relevant view in this case.

26 By which I mean, goods that are able to be apprehended and appreciated by the subject.

27 McMahan notes that the Harm-Based Account (and presumably the Time-Relative Interest Account by implication) is ordinarily associated with the denial of a moral distinction between killing and letting die—but it may be that other accounts of the wrongness of killing do permit such a distinction.

28 The latter clause is important—McMahan does not deny that some killings can be more or less wrong, but that any variation must depend on factors other than the value of the victim or her life—for example, number of killings, motivation, intention, and so on.

29 We explicate exactly what this could mean shortly.

30 This fits neatly with the conception of human dignity as a “rank” that requires respect—this rank could explain the equal wrongness of killing, among other things. Zylberman (2016) has a helpful summary of the issues raised here.

31 The legal codification of the civil and political rights declared in the Universal Declaration of Human Rights.

32 McMahan writes that it is “permissible to sacrifice an animal for the greater benefit of other animals or persons … In the case of persons, however, we believe that it is wrong to kill one person as a means of preventing the killing of a greater number of others” (2002, 246–7).

33 “We hold these truths to be self-evident, that all men are created equal, that they are endowed by their Creator with certain unalienable Rights, that among these are Life, Liberty and the pursuit of Happiness.” In his most famous speech during the Civil War to end slavery, the Gettysburg Address, Lincoln relies heavily on this theme.

34 The account also fits neatly with Kant’s account of human dignity and worth.

35 Clearly, it is possible (in many cases, likely) that someone *is* consciously harmed by these actions—but this is not *necessarily* so.

36
Rodger, Blackshaw, and Miller (2018) offer some further examples in the context of the beginning of life.

37 Jonathan Haidt’s *The Righteous Mind* is a good introduction to a kind of common sense, pluralist morality that admits questions of sacredness and respect even in the absence of harm; see Part II in particular. Haidt shows how these notions are endemic to our thinking whether or not we are religious—ideas of respect and sacredness are a part of everyday moral thought, even if increasingly by the former label.

38 A reviewer points out that assisted suicide or euthanasia might be thought compatible with respect. This helpfully demonstrates how the respect account of the wrongness of killing is precisely that to which pro-lifers subscribe: they typically believe that killing oneself or another person is wrong even when solicited. All I need is for this theory to be plausible on the pro-life view. Even if one supposes that suicide and euthanasia are not necessarily disrespectful, the premise could easily be modified: killing someone without their consent is necessarily a failure of respect.

39 The most obvious is that doing so is metaphysically impossible, which—as I have argued—may be the case for many miscarriages.

40 Just as, for example, intending to kill may be as serious as killing, even if one fails.

41 It goes without saying that pro-lifers do not typically fail to prevent miscarriages deliberately, or out of a lack of respect, but out of a lack of resources.

42 There are a few fringe cases, such as in *Re A (conjoined twins)*, which do not make a significant difference to this argument.

43 This is not to say that I condone the widespread greed that also characterizes many people in the developed world. I do think that we have a duty to give much more to save lives in the developing world than we currently give; I just do not think that duty necessarily overrides any duty or permission to spend resources on goods that do not save lives.

44 Again, of course, by contrast, killing a woman of the same age to promote equality of women is not permissible.

45 As Greasley (2017) has compellingly argued.

46 For full transparency, I have no hesitation in saying that I believe a relatively compelling argument can be given for this as well.

47 In short, because the norms on saving lives are governed by many factors other than human equality: and these other factors are present in such cases. This does not impugn the ultimate equality between the embryos and the child.

48 Greasley offers a comprehensive analysis of the significance of this distinction—while letting die is frequently permitted in many jurisdictions, killing has far more stringent restrictions. Hence, since abortion appears to be killing rather than letting die, the restrictions on it are far more stringent than they would be if abortion were merely a failure to rescue, as Thomson’s violinist experiment might imply.
